# Man-animal relationships in Central Nepal

**DOI:** 10.1186/1746-4269-6-31

**Published:** 2010-11-04

**Authors:** Usha Lohani

**Affiliations:** 1 Central Department of Zoology, Tribhuvan University, Kathmandu, Nepal

## Abstract

**Background:**

Nepal is small in size but rich in bio-cultural diversity. The rugged terrain of the country is home to a number of unique assemblages of fauna, some of which are endemic. Not only faunal resources the country also harbors some very ancient populations whose interrelationship with these diverse faunal resources is very intimate and thus demands scientific study. Animals play important role in both material and spiritual spheres of their life. There are more than hundred groups of such populations in the country and the group Tamang is one of these. The present paper studies Tamang-animal relationships in central Nepal.

There is a growing trend of scientific ethnozoological studies all across the globe, but this field is yet in its infancy in Nepal. The country is losing important fauna as well as ancient human cultures at the advent of development processes. As a result, ethnozoological knowledge is also teetering on the brink of extinction.

**Methods:**

Ethnozoological data were collected by applying different participatory approaches techniques such as semi-structured interviews, participatory rural appraisal, key informant interviews and focus group discussions.

Quantitative data were obtained by employing a household level questionnaire survey. Data were collected from the period of September 2004 to August 2005.

Most of the animals were identified up to the species level with the help of standard taxonomic keys.

**Results:**

The Tamang community treasures knowledge on various uses of 41 genera belonging to 28 families. Out of total number of animals, 14.6% belong to the Invertebrate group and the rest to the Vertebrate group. Of the total uses 58% fall in the food and medicinal use category, 16% in the magico-religious use category, 18% in the category of omen indication, and 2% each in the categories such as weather forecasting, trophy, ethnomusical and taboos.

**Conclusions:**

The Tamang maintain strong ties with animals both at a material as well as spiritual level. While some animals are the sources of important traditional medicines, others are omen indicators and weather forecasters. High priority should be given in conservation of those animals which are of high consensus value to the community.

## Background

Mankind's relationship with animals has been intimate right from the beginning of civilization. Animals have been playing an important role in human economy, culture, religion and magico-religion. Human beings, especially the ancient or the ethnic people, in turn have been maintaining a healthy natural environment by making prudent use of the surrounding biological resources. There is a recent trend of erosion of this type of age-old man-animal relationship because of the loss of animal resources primarily due to rapid urbanization. Nevertheless, the world's high biodiversity areas are found in the land of ancient populations even now [1].

Animals, whether domestic or wild, have always been providing a number of goods and services to human beings. Wildlife provides protein and other necessary food materials to the rural communities in 62 countries of the developing world [2-4]. Besides this, animal and animal derived products have always been sources of traditional and modern medicines. Even now, animal parts and products such as bile, excreta, urine, endo and exo-skeletal parts, skin and viscera all form important parts of the traditional pharmacopoeia across the globe [5-13]. So far as modern medicine is concerned, it is reported that more than half of the world's modern drugs are of biological sources [14,15]. Of the 252 essential chemicals that have been selected by World Health Organization, 8.7% come from animal sources [15].

Animals occupy an important position in culture and religion of traditional societies all over the world. In this context, many of the useful animals are given sacred status because of their important roles in human culture and religion. Examples could be cited of various domestic cow species which are worshipped by the traditional Hindu societies on a regular basis in recognition of their values to mankind [16]. Not only this, even products such as excreta and urine of these animals are smeared in the floor areas of their houses with a belief that these products would sanctify their dwellings. Similarly, certain body parts such as the horn, hide, tail and feathers of some other species of animals are used in their religious rituals as well.

Traditional societies use a number of animals in their magico-religious sphere. Normally animal parts and products such as the exoskeleton, bone and glandular secretions are used as pendants and amulets to ward off the perceived evil spirits. In many places it is a common practice to offer animal sacrifices to appease specific deities and ancestral spirits. The sacrificial offerings are regarded as gifts to the deities that are supposed to maintain health and general well-being of those involved in the process [17,18].

Some animals indicate omens and forecast future weather conditions to the traditional societies of different places [19-21].

The Eastern Himalaya - a region spanning Nepal, Bhutan, north-eastern India, Tibet and far-north of Burma, is known as a global biodiversity hotspot. Nepal, though small in size is thus rich in biodiversity. The presence of valleys, gorges, mountain cliffs, rivers and rivulets within a small area of the country has further contributed to a number of specific niches, which harbor unique assemblages of flora and fauna. Such specific habitats also harbor quite a few numbers of endemic species of fauna and flora. The country though small in size is thus home to an estimated 300 spp. of mammals, 977 spp. of birds, 176 spp. of reptiles, 105 spp. of amphibians and 269 spp. of freshwater fish [22].

Co-existing with such unique and varied flora and fauna in the difficult mountain terrain are a number of ancient groups of people popularly called ethnic groups. Like other ancient populations of the world, these people too have been interacting with the surrounding biological resources through ages to fulfill their material and spiritual needs.

Much of the historical and contemporary indigenous knowledge of numerous ethnic groups around the world is at as much risk of being lost; as is the case with biodiversity [23]. Nepal is not an exception to this ground reality. Deforestation is resulting in an increasing loss of habitats for birds, wild animals and reptiles. IUCN reported that 24 species of mammals, 9 species of reptiles, 27 species of birds, 2 species of insects and 13 species of plants have become endangered in Nepal till now [24]. Also, there is a gradual loss of varied human cultures, where such knowledge is deeply rooted. Very low population share of 0.05% and 0.02% of two of such ethnic groups namely Pahari and Jirel respectively is indicative of their fragile and vulnerable status [25].

In view of an increasing relevance of the ethnobiological knowledge all across the globe, scientific study of such valuable knowledge has just made its beginning in Nepal. Ethnozoogical research in the country has often been much neglected in comparison to ethnobotanical research. That is why a number of scientists in the country have emphasized for a strong need for the scientific study and documentation of such a valuable and ever relevant body of ethnozoological knowledge [26]. Hence, the present work is aimed at studying the interrelationship that exists between animal resources and a small group of people belonging to the ethnic group Tamang from the Central Mountainous region of Nepal. A database of ethnozoological knowledge can also serve as baseline data for further research in the country. Animals that have more use values are often the most exploited ones and thus the most threatened [27]. The present work would also draw attention of the concerned authorities on keeping such animal resources in the highest priority of conservation. Also, since higher percentages of animals in this study are of food and medicinal value, the work would be all the more important especially at a time when modern medicine is turning towards animal parts, products, and secretions to cure humans.

## Methods

### Study area

Nepal is a small mountainous country with an area of only 147,181 sq km expanding along the central and eastern Himalaya. The country is 885 km long from east to west and 193 km broad from south to north. Within a horizontal expanse of less than 200 km the altitudinal height of the country in the south north direction varies from less than 100 m to 8848 m (Mt. Everest). Sharp altitudinal variations within a small expanse of land have given rise to specific land pockets and varied climatic conditions. All these conditions in turn have contributed to the diverse and unique assemblages of flora and fauna. Biodiversity in the country has emanated from other factors as well besides altitudinal variations. The country is located along the central and eastern Himalaya encompassing two zoogeographical realms - the Palaerctic and the Oriental. The northern part of the country harbors Palaerctic fauna and flora whereas the southern part is the homeland of Oriental biological elements. The central part or the part between these two realms however, shows mixed faunal and floral elements of both the realms. This part of the country harbors many of the ethnic groups of the country. The group Tamang is one of the ethnic groups of the region

**Figure 1:**
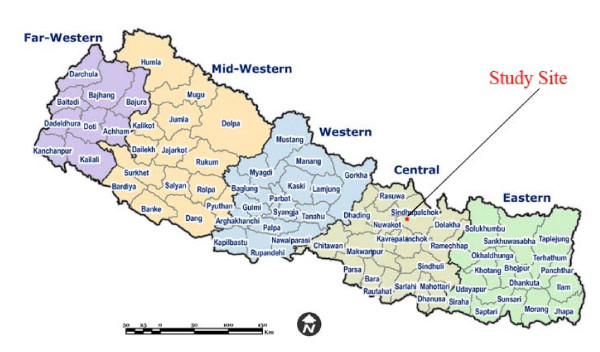
**The study site in the map of Nepal**.

The present research work was conducted on the Tamangs of the two villages in the central mountainous part of the country. These villages belonged to the two Village Development Committees (hereafter called VDC) Thumpakhar and Thulopakhar of the district Sindhupalchok of the region (Figure 1). The work was carried out by the author as a part of her PhD research project from September 2004 - January 2005.

The majority of the Tamangs are scattered in the high altitude areas between 1500 meters to 2500 meters in the central mountainous region of the country. Of the two study VDCs Thumpakhar holds just 737 Tamang populations whereas the other VDC Thulopakhar harbors 2370 of them. The Tamang constitute 5.64% of the total population of the country [25]. They believe that they originally came from Tibet. They speak Tibeto Burman language and follow Buddhist religion. In their language "Ta" means horse and "mang" means trader indicating that they were horse traders in the ancient time [28]. Like other ethnic groups of the country, Tamangs also used to hold "kipat" - a kind of land tenure system where land management was carried by various clans of their own until a few decades ago. This system is now replaced by a modern system of land tenure with taxing arrangements. The Tamangs show respect towards surrounding biological resources. They do so in recognition to the role these resources have been playing in maintaining the ecological balance of the vicinity. One of the examples where their reverence towards the resources can be clearly understood is "Bhumi Puja" where "mother earth" is worshipped in the name of a deity. Such worship or "puja" is usually performed once a year where the animal sacrificial activities become important part of the ceremony. These people also show respect and thus worship their ancestral spirits (Shamanism). Normally "Bompo" the Tamang priest propitiates the spirits whenever necessary. The Tamangs have a tradition of choosing one of the senior members from their community as their leader. The leader of the village is called "mulmi" who takes important decision for the entire community and even settles minor disputes in the village. Most of the Tamangs are non-vegetarians and thus raise poultry and goats in their house. Besides poultry and mammals, they also relish smaller animals especially amphibians such as frogs and toads. But while eating these animals they have to abide by certain traditional rules. These rules restrict cooking of these animals inside the house. They can however, bring already cooked meat of these animals inside the house and eat it [28]. Similar rules have to be followed by those who wish to eat pork and buffalo meat in their house.

### Socioeconomic profile of the community

**Figure 2:**
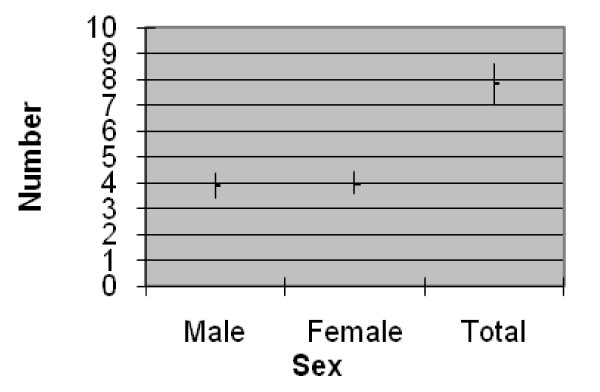
**Average family size together with average male and female number in Tamang community**. Source of data is questionnaire survey (n = 81). Mid-points in the vertical lines in the figure denote average values, whereas the lines represent confident limits of the means at 95% significance level.

**Figure 3:**
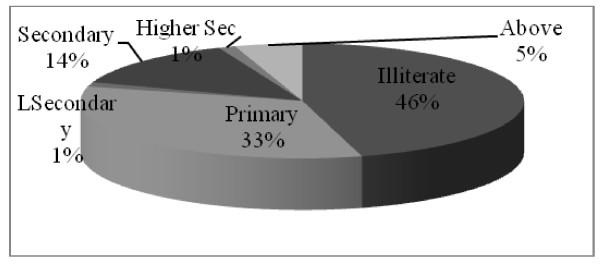
**Education status of Tamang group based on questionnaire survey (n = 81)**. Illiterate = who cannot read and write, Primary level = grade one to five, Lower secondary level = grade six and seven, Secondary level = grade eight to ten, Higher secondary level or Intermediate level = grade eleven and twelve and above = bachelor and higher level.

**Figure 4:**
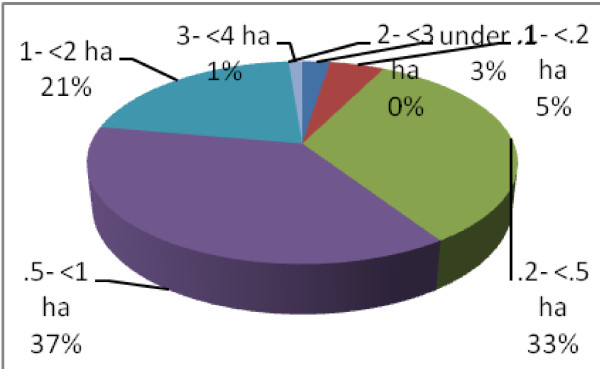
**Per family land holding in hectare in Tamang group (n = 81)**.

a. Family size: Family size of the community is 7.8 +- 0.8 (Figure 2) which is greater than the district average of 5.06 [29].

b. Education status: 54% of the Tamangs are literate out of which almost 33% have attained only primary education (Figure 3). The literacy rate is higher than that of the district average of 40.6% and almost equal to the literacy rate of the country which is 54.1 for the year 2001[29].

c. Economic activities: The Tamangs primarily live upon scale agriculture for their livelihood. Average land holding size/family is 0.67 ha. People (in percentages) with different land holding sizes are clear from Figure 4. Main crops are paddy, wheat, maize, millet and potato. Paddy dominates other crops with its highest per year production yield.

**Figure 5:**
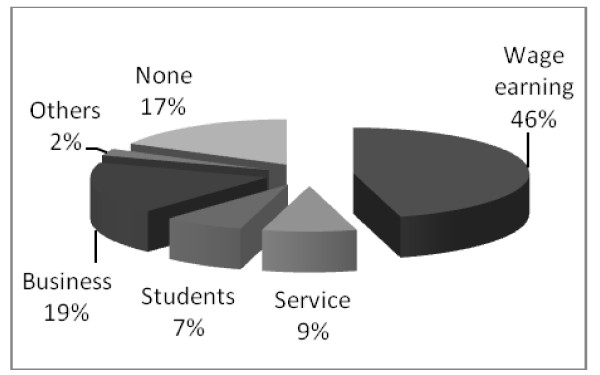
**Percentages of people with other economic activities besides agriculture (n = 81)**.

**Figure 6:**
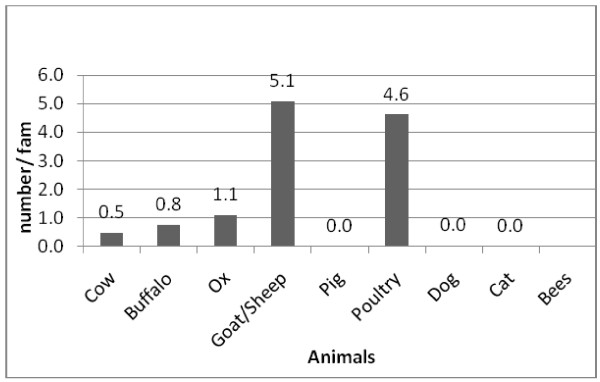
**Per family livestock number (n = 81)**.

Other economic activities of the community besides agriculture are wage earning (46%), business (19%), service (9%) and other unspecified sectors as illustrated by Figure 5. Among these 7% of them are students and 17% are without other economic activities besides agriculture.

d. Animal husbandry: Livestock unit/family (only cow, ox, buffalo and sheep/goat are calculated) is 3.8 +- .07 (1 cattle = 1 unit, 1 buffalo = 1.5 units, 1 goat/sheep = 0.2 unit). Per family livestock composition of the group is illustrated by Figure 6. Livestock raising is integral to their agriculture based economy. It is linked with agriculture for there is constant flow of energy in the form of manure from livestock to the farm. Oxen are used to plough the rugged terrain of the area. About 59% of the families have raised oxen for this purpose. Livestock also provides protein to them. Almost 89% of the respondents have kept goats in their house. Distress selling of the goats is a common practice. Only 49% of the families are involved in buffalo rearing for milk, meat and manure. Cows are kept by only 28% of them. It is interesting to note that not a single family has reared pigs for pork eating is a taboo among the Tamangs. Those who wish to eat pork will have to manage to cook it elsewhere instead of cooking it in the regular kitchen of the house.

### Data collection

Both qualitative and quantitative methods were adopted in the present study. Quantitative data were obtained by employing a household level questionnaire survey. For this, 13% households (81 households) were selected by a simple random sampling technique out of 621 households. The sample was thought to be a representative one for a culturally homogenous group. The survey was conducted to find out information on family size, land holding size and number and types of livestock raised, literacy status and other economic activities. The head of the household was interviewed for the survey. The most preferred livestock to the community was identified from the data on animal husbandry and the economic condition of the group was assessed from the socioeconomic data.

Ethnozoological data were collected by applying different techniques of participatory approaches such as semi-structured interviews, participatory rural appraisal, key informant interviews and focus group discussions.

Most of the animals were identified up to the species level but a few could be identified only up to generic level. Larger animals were identified according to the folk description of each specimen and from the pictures shown to them. Standard taxonomic keys of taxonomists from home and abroad were used to make correct identification of the animals [30-35]. Ethnozoological data were presented in a tabular form under the headings such as common English name, zoological name, local name and a brief ethnozoological note. Popularity of the animal uses was assessed from the data on consensus indices.

## Results and discussion

### Ethnozoological data

Table 1 given in the article shows the animal use patterns of the Tamangs. Ethnozoological knowledge of the studied community reveals various uses of 41 genera belonging to 28 families. Out of total number of animals, 14.6% belong to the Invertebrate group and the rest to the Vertebrate group. Of the total uses 58% fall in the food and medicinal use category, 16% in the magico-religious use category, 18% in the category of omen indication, and 2% each in the categories such as weather forecasting, trophy, ethnomusical and taboos.

**Table 1:** Animals with their uses in the study group Tamang

Common name/Family/Sub-family	Scientific name	Local name	Uses	Use category	Consensus index
Earthworm **Megascolecidae**	*Pheretima *spp.	"Gadeula"	Earthworms are ground and the mixture is filtered. The filtrate is administered orally to children suffering from high fever due to measles and chicken pox.	M	xx

Honey bee/ **Apidae**	*Apis *spp.	"Mauri"	Honey is used in cuts, wounds, burns, gastritis and urinary problems. Bees are allowed to sting heart patients to cure heart problems. (Bee venom therapy)	M	xxx

Crab **Potamidae**	*Himalayapotamon atkinsonianum *Woodmason, 1871	"Gangato"	Crabs are used in a number of ways. These are administered orally to small children to stop bedwetting (enuresis). Cooked or roasted crabs are eaten to sharpen memory and to treat gastritis.	M	xx

Wasps &Hornets **Vespidae**	*Poliste*s spp. *Vespa *spp.	"Barulo" "Aringal"	These are fried and eaten. Broods are preferred to the adults in taste.	F	xx

Slug **Limacidae**	*Limax *sp.	"Chiplekira"	Slugs are eaten raw either mixing with a ball of clarified butter or mixing with hot milk for nutrition usually by patients of chronic illness like TB. Slugs are also used to join the fractured bone. For this purpose these are ground to fine paste and the paste is then applied at the site of the fracture.	M, F	xxx

Hill Trout/ **Cyprinidae**/Schizothoracinae	*Schizothorax plagiostomus *(Heckel, 1838)	"Sun Asala"	Alimentary canal along with the inner contents is cooked and eaten by persons suffering from gastritis. Bile juice has a medicinal value and is purported to cure a number of ailments along with gastritis.	M, F	x

Nepalese Minnow/ **Psilorhynchidae**	*Psilorhynchus pseudecheneis *(Menon & Dutta, 1964)	"Tite Maccha"	This fish is eaten to cure high fever and to maintain free passage of urine in case of its obstruction due to some unknown reasons. Bile is administered orally to cure fever.	M, F	x

Carps/**Cyprinidae/** **S fam. Cyprininae**	*Tor tor *(Hamilton, 1822)	"Sahar"	Bile juice is administered orally in cases of high fever.	M	x

Liebig's frog/**Ranidae**	*Paa liebigii* (Günther, 1860)	"Man Paha" "Kalo Paha" "Beng"	It provides a good source of protein and nourishment to the local people. A small piece of dried skin is soaked in water and rubbed on stone to obtain a paste, which is then applied to wounds and burns.	M	xx

Paha/ **Ranidae**	*Paa polunini *(Smith, 1951)	"Sano Paha"	Dried frog is ground to a smooth paste and is given to children suffering from diarrhea and dysentery. The paste is also used to reduce scars in the skin caused by wounds and burns.	M	xx

Snake/ **Colubridae**	----------	"Sarpa"	It is believed that if a snake crosses the road before setting on a journey, it is inauspicious. Meat of any species of snake is eaten to improve the eyesight. Fat from cooked snake is believed to cure even cancerous wounds.	OI M	x

Lizards/ **Gekkonidae**	*Hemidactylus *spp.	"Mausuli", "Cchipkili"	Charms are made out of bones of the lizards and it is believed that these drive evil spirits away.	MR	xx

Fowl/ **Phasianidae**	*Gallus gallus* (Linnaeus, 1758)	"Kukhura"	Chicken soup is considered to be nourishing food especially for women just after delivery. Fat is used to give massages for the treatment of sprains and strains. Both incidents such as crowing of the cock at a time other than morning and crowing of hen are thought to be bad omens.	F, M, OI	xxx

Starling/ **Sturnidae**	*Sturnus *spp.	"Saraun"	Raw blood is eaten to be cured from chronic blood dysentery. The cooked bird may also cure diarrhea and dysentery.	M	x

Whistling Thrush/ **Muscicapidae/** **Turdinae**	*Myiophonus caeruleus *(Scopoli, 1786)	"Kalchaude" "Kalungo"	Cooked flesh is given to women suffering from menstrual irregularities and infertility. Its meat is also thought to lessen pain during delivery.	M	xx

Crested serpent eagle/ **Accipitridae**	*Spilornis cheela *(Latham, 1790)	"Kakakul"	Cooked flesh is given to the person suffering from diarrhea and vomiting. Charms are made out of bones, claws and head part and given to wear to children for their general well being. Weather forecast - If the bird makes shrill sound, it predicts rainfall in the near future.	M MR WF	x

Eurasian Griffon/ **Accipitridae**	*Gyps fulvus *(Hablizl, 1783)	"Giddha"	It is used as an omen indicator. If it is found to be hovering above a particular house, it is believed that there is sure going to be sad demise of one of the family members. In other words, the Griffon predicts death of one of the persons of the area.	OI	xxx

House Crow/ **Corvidae/** **Corvinae**	*Corvus splendens *Vieillot, 1817	"Kag"	If a crow crows early in the morning facing a house, it indicates some news for the family. But if it crows perching on a bare twig, it indicates bad news for the family.	OI	xxx

House Swallow/ **Hirundinidae**	*Hirundo rustica *(Linnaeus, 1758)	"Gaunthali"	There is a belief among the rural folk that if anybody kills the swallow, he or she is sure to suffer from leprosy. This kind of notion places a taboo on the killing of the swallow.	S	x

Fish owl/ **Strigidae**	*Ketupa *spp.	"Hoochil"	This bird produces different tones of sound in different time periods. It is believed that if it produces laughing sound, it indicates death of a person in the nearby area.	OI	xxx

Dove/ **Columbidae**	*Streptopelia *spp.	"Dhukur"	Flesh is cooked and eaten for its nutritive value.	F, M	x

Horned owl/ **Strigidae**	*Bubo bubo *(Linnaeus, 1758)	"Hapsillo"	This bird acts as omen indicator. If it makes a sound like "hoon-hoon", it indicates bad news. On the other hand, if it makes sound like "pau-pau" some news is indicated, but not bad news.	OI	xx

Rabbit/ **Leporidae**	*Lepus nigricollis nigricollis*(Cuvier, 1823)	"Kharayo"	It is believed that fresh blood of rabbit lowers high blood pressure.	F, M	x

Sheep/ **Bovidae**	*Ovis ammon *(Linnaeus, 1758)	"Argali"	Fat is applied to scars made by wounds and burns, causing the skin texture to become smooth and tender.	M	xxx

Domestic cow/ **Bovidae**	*Bos *spp.	"Gai"	Bile is taken orally to cure stomach ache, gastritis and diarrhea.	M	xxx

Buffalo/ **Bovidae**	*Bubalus *sp.	"Bhainsi"	Fat is used to cure scars and cracks in the skin. Bile from the gall bladder is consumed to cure gastritis.	M	xxx

Wild Yak/ **Bovidae**	*Bos grunniens mutus* (Przewalski, 1883)	"Yak"(Male) "Chourigai" (female)	The tail is used in faith healing.	MR	xxx

Sloth bear/ **Ursidae**	*Melursus ursinus *(Shaw, 1791)	"Kathe bhalu"	Bile juice from the animal is boiled in water and the soup is taken orally to cure gastritis, other stomach problems and TB. A bile and cow milk mixture, prepared by mixing a few drops of bile in about 200 ml of milk, is given to cure asthma. The patient's body is massaged with its fat to lessen muscular pain. Charms are made out of bones and worn to drive off evil spirits.	M MR	xx

Barking deer/ **Cervidae**	*Muntiacus muntjac *(Zimmermann, 1780)	"Ratuwa"	A kind of musical instrument called "Damphu" is made out of its skin. Raw blood is taken orally to cure dysentery.	EMUS M	xx

Deer/ **Cervida**e	*Cervus unicolor *Kerr, 1792	"sambar" or "Jaratyo"	Faith healers use horns of the animal in their treatment mechanism. These are also kept in the rural houses as decorative items. Bones are boiled to make a thick and concentrated soup, which is taken orally to cure various ailments like rheumatism, backache and etc.	MR T	xx

Deer/ **Cervidae**	*Axis axis *(Erxleben, 1777)	"Chital"	It is valued as a food.	F	x

Musk deer/ **Cervidae**	*Moschus chrysogaster *(Hodgson, 1839))	"Kasturi Mriga"	Musk or "kasturi" is used in making medicines. Local people use musk to make charms, which are normally worn to ward off their enemies.	M, MR	xxx

Porcupine/ **Hystricidae**	*Hystrix indica *(Kerr, 1792) *H. brachyura *(Linnaeus, 1758)	"Dumsi"	The alimentary canal is boiled along with its contents and the soup thus prepared is taken orally to cure asthma. Meat serves as good source of protein to the villagers. Bile juice is taken orally to cure typhoid or applied externally in the treatment of wounds.	M, F	xxx

Jackal/ **Canidae**	*Canis aureus *(Linnaeus, 1758)	"Syal"	Jackal flesh is mixed with millet or locally produced cereal and yeast to produce alcohol. Alcohol released by fermentation of the mixture is trapped by the distillation process. This alcoholic beverage, called "Syalko raksi", is very popular among the ethnic group. It is purported to be of high medicinal value. It is used in massages for the treatment of body aches (gout and arthritis) or even taken orally for relief.	M	xxx

Dog/ **Canidae**	*Canis *spp.	"Kukkur"	The crys of a dog indicate a bad omen.	OI	x

Red fox Indian fox/ **Canidae**	*Vulpes vulpes *(Linnaeus, 1758) *V. bengalensis *(Shaw1800)	"Phyauro" "Lomri"	It is a common belief amongst the rural folk that a howling fox indicates a bad omen and predicts the death of a person in the village.	OI	x

Cat/ **Felidae**	*Felis bengalensis *Kerr, 1792	"Biralo"	If a cat crosses the road before the onset of journey, it is cancelled because of fear of bad luck in future. It is considered to be bad omen. A black cat is thought to be a symbol of a witch.	OI	x

Tiger/ **Felidae**	*Panthera tigris tigris *(Linnaeus, 1758)	"Bagh"	Tiger milk is supposed to prevent a fire from further propagation. Tiger bones, claws and whiskers are used in making charms, which are thought to bring strength and vigor to the wearer and frighten the enemy.	S, MR	x

Hanuman Langur **Cercopithecidae**	*Semnopithecus entellus *(Dufresne, 1797)	"Langur"	Cooked meat reduces joint pain.	M	x

*Abbreviations used in use category column: *F: food, M: medicinal, MR: magico-religious, OI: omen indicating, S: superstitious, WF: weather forecast, EMUS: ethnomusical and T: Trophy

*The consensus index reflects the spontaneous quotation frequencies for different remedies*: x: use quoted by less than 10% of the informants; xx: use quoted by more than 10% and less than 40% of the informants; xxx: use quoted by more than 40% of the informants.

#### Food and medicinal animals

Since there is overlapping of food and medicinal values in case of many animals, both these use categories are kept under single heading of "Food and medicinal animals". It is found that 68% of the animals (n = 81) both vertebrates and invertebrates are of food and medicinal values. Animals such as *Apis *spp., *Schizothorax *spp., *Paa *spp., snakes spp., *Gallus *sp., *Lepus *sp., *Ovis *sp., *Histrix *spp. have both food and medicinal values. Animals such as slugs, *Pheretima *spp., *Sturnus *spp., *Myophonus *spp., *Spilornis *spp. are of only medicinal value and are eaten either cooked or raw. Wildlife has been playing an important role in minimizing the existing protein gap in different parts of the world. It has been reported that rural populations from at least 62 countries have been obtaining protein and other essential food supplements from wildlife [36]. Overlapping of food and medicinal uses is a common finding from India and other parts of the world [37-43]. Such animals with dual role are all the more important for these not only heal the ailment but also provide nourishing food item to boost up the immune system. One of the animals with double role among the invertebrates is *Pheretima *spp. Mass production of such animal spp. will form important source of ethnomedicine as well as protein supplement to the people. Healing with earthworm is an ancient practice and is still in popular among many traditional societies in Asia [39,40].

Both wild and domestic animals are sources of traditional medicines. In this study, we found that parts and products of four domestic animals such as domestic cow, buffalo and sheep are used in healing while the rest are from the wild animal resources. Domestic animals are sources of traditional medicine to both human and livestock in a number of Mediterranean countries besides Nepal [44].

Method of preparation and application of zootherapeutics vary according to the animals and the ailments to be treated. In the cases of animals such as *Schizothorax *spp. and *Hystrix *spp. targets are made on their ingested food rather than on their parts or products. These animals are killed merely for their alimentary canal that remains packed with ingested fresh plant materials such as algae and herbs which are then boiled in water and the soup is consumed to get rid of asthma and other respiratory problems. Porcupine (*Hystrix *spp.) is believed to eat Himalayan herbs of medicinal value. This is a unique case of phytotherapy where animals are used to obtain targeted medicinal herbs.

Raw blood of a bird (*Sturnus *sp.) is considered to be of tremendous zootherapeutic potential in curing blood dysentery. It is obvious here that the Tamang try to compensate blood loss due to dysentery by the intake of avian blood. There are also reports of healing with raw blood from other parts of the world [39].

Bile from mammals such as the domestic cow (*Bos *spp.), buffalo (*Bubalus bubalis*), sloth bear (*Melurus ursinus) *and porcupine *(Hystrix *spp.) is used in burns, wounds, cuts, gastritis and stomach related problems, typhoid, fever and asthma. It has been reported that the alkaline nature of bile helps in regulating the body temperature [45]. In many Asian countries, bear bladder is considered to be very useful in digestive illnesses. Bear bile has been very popular in Traditinal Chinese Medicine for thousands of years [46].

Fat of mammals is usually used as a topical ointment to heal cracked skin, burns, rheumatism and arthritis. Similar types of applications of mammalian fat have also been reported in India and abroad [47,48].

Alcohol prepared by fermenting the mixture of jackal flesh and local cereals, also called "Jackal alcohol", is very popular with high a citation frequency and is given to patients with gout and arthritis. This is a unique case of zootherapy prevalent in Nepal. There are no known reports of similar kinds of zootherapy from other parts of the world.

#### Animals in a Magico-religious sphere

Animal parts and products such as bones and claws of the wall lizard (*Hemidactylus *spp.) and serpent eagle (*Spilornis cheela*), bones, claws and whiskers of tiger (*Panthera tigris*), and musk of musk deer (*Moschus *spp.), are used in making charms and tied round the neck and arm to ward off perceived "evil and disease causing elements". Faith healers use the tail of Himalayan cow- Yak (*Bos *spp.) and horns of deer (*Cervus unicolor*) in their faith healing therapy. Tiger milk (*Panthera tigris) *is believed to prevent further propagation of fire in the village. In this way, the Tamang animal relationship is observed not only at the material level but also at a spiritual level. Relationships of the tribal people with the animals at the spiritual/cultural level is reported from different parts of the developing world [49,50].

#### Animals in omen indication, and weather forecast

Crossing of the road by snakes and cats of any species indicates a bad omen. Similarly, flying of *Gyps fulvus *above the house predicts death of one of the members of the house. Unusual sounds like "**hoon-hoon**" of *Bubo bubo *and the laughing sound of fish owl (*Ketupa *spp.) and howling of fox (*Vulpes vulpes*) predict bad news for the family of the vicinity. The villagers believe that such a sound could even lead to the death of a close relative of the family. Domestic animals such as cats and dogs indicate omens which could be good or bad depending upon their particular activity at different times. When the Serpent eagle (*Spilornis cheela*) makes a shrill sound instead of its usual sound, it indicates rainfall in the near future. Similar reports of weather forecasting by birds and insects are available from India and abroad [21,51].

#### Ethnomusical animals and animal parts used as trophy

Skin of *Muntiacus muntjac *is used in making a kind of musical instrument called "*Damphu*" by the community. Deer horn *Cervus unicolor *is kept in the house of some of the Tamangs as an object of decoration.

#### Taboos

It is believed that if someone kills the swallow (*Hirundo rustica*), the killer is sure to suffer from leprosy. Similar kinds of taboos are reported from other parts of Nepal. It is a taboo to kill the female animal (especially gravid females) among the inhabitants in the northern trans-Himalayan part of the country [52]. This kind of taboo regarding killing of the female animals ultimately contributes to their conservation. These activities could even be referred to as ancient peoples' built-in resource conservation mechanism.

Commercial utilization of some of the new drugs of animal origin could raise poor socioeconomic status of the communities from where the knowledge originated. Priorities should be given to research on such animals. Sustainable use of some of the endangered animals is possible only when their number increases. Possibilities of in-situ conservation of such animals need to be explored. Both systems of knowledge such as traditional and modern should be integrated for the formulation of appropriate policy regarding conservation of such animals.

## Conclusion

The Tamangs harbor a rich body of ethnozoological knowledge because of an intimate relationship over a long period of time. Animals are integral to their culture, religion, magico-religion and traditional pharmacopoeia. In other words, Tamang-animal relationship can be observed both at material and spiritual levels. Body parts/tissues such as alimentary canal, gall bladder, fat, bone, blood, claws, whiskers, are normally used in healing. Endo and exoskeletal parts are usually worn as pendants and amulets to ward off perceived evil elements. Even when the Tamangs have access to modern health care modalities, some of the zootherapeutic practices still remain prevalent in the village. This is a clear indication of their faith in the animal-derived medicine. They still prefer to use alcohol obtained from the fermented mixture of jackal flesh and locally grown cereal to treat gout and arthritis. Similarly, animals such as slug spp., earthworm spp., frog spp., and some of the bird spp. are sources of ethnomedicine to the group till now. This is not limited to wild animals - even domestic animals such as cattle, buffalo, sheep and goats provide useful zootherapeutic remedies to them. The Tamangs hold taboos of killing some animal spp. This type of notion towards animals is often of considerable significance for it contributes to their conservation.

Human-animal relationships at all levels needs to be maintained for the overall development (both physical and mental) of human beings. Conservation of not only biodiversity but also of cultural diversity is necessary for this. Advanced research on animals of excessive medicinal values such *as Pheretima *sp, slugs, *M. caeruleu*s, *Hystrix *spp., *C. aurius*, *M. ursinus *can lead to new sources of drugs.

## Competing interests

The author declares that they have no competing interests.
